# Fabrication of High-Load Curcumin Emulsion Gels via Heat-Induced Confined Recrystallization and Polysaccharide–Beeswax Oleogel Synergy: Oxidation Protection and Gastrointestinal Delivery

**DOI:** 10.3390/foods15213918

**Published:** 2026-11-02

**Authors:** Jinyu Miao, Mengjie Zhu, Liqiang Zou, Wei Liu

**Affiliations:** 1 State Key Laboratory of Food Science and Resources, Nanchang University, No. 235 Nanjing East Road, Nanchang 330047, China; miaojy2023@163.com (J.M.); mj.zhu4429@outlook.com (M.Z.); zouliqiang2010@163.com (L.Z.); 2 International Institute of Food Innovation Co., Ltd., Nanchang University, Luozhu Road, Xiaolan Economic and Technological Development Zone, Nanchang 330200, China; 3 National R&D Center for Freshwater Fish Processing, Jiangxi Normal University, Nanchang 330022, China

**Keywords:** chitosan, hyaluronic acid, fish oil, digestion, stability

## Abstract

Curcumin exhibits anti-inflammatory, antioxidant and lipid-regulating activities, yet it suffers from poor water and oil solubility, low loading capacity in conventional emulsions and facile recrystallization–sedimentation at high concentrations. This work presents a “polysaccharide–interfacial synergistic oleogel-mediated crystal interfacial adsorption” strategy. The oil phase composed of fish oil, coconut oil and beeswax undergoes heating–cooling treatment to induce confined recrystallization of curcumin and beeswax inside droplets, forming an oleogel matrix for crystal entrapment. The aqueous phase employs chitosan (CS) or electrostatically assembled chitosan–hyaluronic acid (CS-HA), with cinnamaldehyde–chitosan imine bonds constructed to build interfacial layers for stabilizing high-loading emulsions. Heating–cooling-treated emulsions achieve >95% curcumin encapsulation efficiencies, versus ~35% for non-heated controls. With increasing beeswax concentration, the onset melting temperature of crystals within emulsions decreased from 171.1 °C (CS-HA, without beeswax) to 98.8 °C (CS-HA + 6%, 6%, *w*/*w*, beeswax), enhancing emulsion gel strength and centrifugal and freeze–thaw stabilities. During in-vitro simulated digestion, the 2% (*w*/*w*) beeswax dual-polysaccharide emulsion (CS-HA + 2%) exhibits optimal digestive stability (79.49%), bioaccessibility (20.77%) and estimated colonic delivery rate (62.45%, ~32% higher than free oil). After 14-day accelerated storage at 45 °C, its primary oxidation products decrease by ~53% relative to free oil. This polysaccharide–oleogel emulsion system combines high curcumin loading, fish-oil antioxidation and colon delivery, providing references for high-dose oral delivery of hydrophobic bioactives.

## 1. Introduction

Curcumin is a hydrophobic polyphenolic compound extracted from the rhizome of turmeric that exhibits various physiological activities, including anti-inflammatory, antioxidant, antitumor, and regulatory effects on glucose and lipid metabolism [1,2]. Epidemiological and clinical evidence suggests that curcumin supplementation can, to some extent, improve the lipid profile in patients with atherogenic dyslipidemia; its lipid-regulating effects are associated with mechanisms such as the inhibition of lipid synthesis, the promotion of fatty acid oxidation, and the reduction in oxidative stress [3]. However, curcumin is extremely poorly water-soluble and has very limited solubility in oil phases (approximately 1.3 mg/mL in soybean oil and 1.1 mg/mL in sunflower oil) [4]. Following oral administration, it is poorly absorbed in the gastrointestinal tract, rapidly metabolized, and has low bioavailability [5]. To improve its oral delivery efficiency, a large number of nanocarriers have been reported; however, the actual loading capacity of curcumin in such carriers is generally low, making it difficult to meet the dosage requirements for the development of health products in the food industry and for *in vivo* efficacy evaluation [6].

On the other hand, accumulating evidence indicates a bidirectional interplay between curcumin and its metabolites and the gut microbiota in the lower intestinal tract, whereby the microbiota biotransforms curcumin into active metabolites, while curcumin in turn modulates the composition of the microbiota [7,8]. Consequently, the targeted delivery of curcumin to the colon, rather than its rapid absorption in the small intestine, may be a key mechanism underlying its intestinal and metabolic regulatory effects [9,10]. Polysaccharide-based colon delivery systems utilise the property of polysaccharides to withstand digestion in the gastric and small intestinal environments whilst being degraded by the microbiota in the colon, making them an important means of colon targeting; however, existing curcumin-loaded colon delivery systems still generally suffer from low drug loading and insufficient structural stability [11,12,13].

In conventional oil-in-water emulsions, high loading of curcumin readily leads to crystallization within the oil phase and subsequent precipitation/sedimentation. Large crystals can also pierce the oil–water interfacial film and induce droplet coalescence, resulting in low encapsulation efficiency and poor system stability. Gelation of the oil phase offers a promising strategy to address these issues: gelators (e.g., beeswax, palm wax, and solid fats) construct a three-dimensional crystal network in the oil phase via heating–cooling to form oleogels, which can inhibit molecular migration and recrystallization of active ingredients, significantly increase the loading capacity of hydrophobic components, retard their release, and improve their bioavailability [14,15]. Meanwhile, the dense oleogel network can impede the diffusion of oxygen and free radicals, thereby retarding lipid oxidation (gel hardness is positively correlated with oxidative stability) [16].

At the interfacial engineering level, polysaccharides offer a mild and efficient approach for the interfacial stabilization of high-loading emulsions. Chitosan is a rare naturally positively charged aminopolysaccharide that can adsorb at the oil–water interface to form a cationic polyelectrolyte film, thereby synergistically stabilizing droplets through steric hindrance and electrostatic repulsion [17]. The aldehyde group of cinnamaldehyde can undergo an in situ Schiff reaction with the amino groups on the chitosan chains to form imine bonds via covalent cross-linking, thereby anchoring the hydrophobic aromatic segments at the oil–water interface. This interfacial in situ reaction requires no external cross-linking agents and proceeds spontaneously under aqueous conditions. It not only significantly enhances the mechanical strength of the interfacial film but also confers potential antimicrobial properties on the interfacial layer, and has been demonstrated to effectively stabilize high internal phase Pickering emulsions [18]. Building upon the single-polysaccharide interface, the introduction of anionic hyaluronic acid enables electrostatic assembly with chitosan adsorbed at the interface to construct a thicker and denser dual-polysaccharide composite interfacial layer, thereby further reducing droplet size, inhibiting aggregation, and enhancing storage stability and *in vitro* digestive stability [19,20]. Furthermore, hyaluronic acid exhibits excellent mucoadhesive properties that can prolong the residence time of the carrier in the gastrointestinal tract [21]. It can also be fermented and utilized by colonic microbiota and modulate microbiota metabolism (e.g., by promoting short-chain fatty acid production), aligning with the design goal of colon-targeted delivery [22]. Based on the above considerations, this study prepared emulsions stabilized by chitosan/hyaluronic acid dual-polysaccharide interfaces and single chitosan interfaces, respectively, to investigate the regulatory effects of polysaccharide interfacial composition on emulsion system construction, lipid oxidative stability, and colonic delivery efficacy.

However, existing oleogel delivery studies mainly construct crystal structures within the oil phase using gelators alone, and the synergy between the oleogel crystal network and the external polysaccharide interface remains poorly understood. It is unclear how the polysaccharide interface cooperates with the dispersed oleogel to inhibit large curcumin crystals, how interfacial composition and beeswax concentration jointly regulate emulsion properties and environmental tolerance, and how this synergy affects colonic curcumin delivery and fish oil oxidative protection.

We hypothesized that heating–cooling-induced oleogelation, combined with the confinement effect of the oleogel network and the chitosan/hyaluronic acid dual-polysaccharide interface, would enhance curcumin loading. Specifically, the oleogel network and interfacial polysaccharides were expected to restrict curcumin crystal growth, while the polysaccharide interface would serve as a barrier to improve the oxidative stability of fish oil and delay curcumin release in the gastrointestinal tract. To test this hypothesis, herein, a “polysaccharide interface–oleogel synergy for regulating crystal” strategy was proposed to construct a high-curcumin-loading colon-targeted emulsion. Fish oil, coconut oil, and beeswax were incorporated into the oil phase, where heating–cooling induced curcumin/beeswax recrystallization to form an oleogel framework confining curcumin crystals. Chitosan (single polysaccharide) or chitosan–hyaluronic acid (dual polysaccharide) was used to build the interface, with cinnamaldehyde–chitosan imine cross-linking providing interfacial stabilization. The effects of beeswax concentration (0%, 1%, 2%, 6%) and polysaccharide interfacial composition on droplet size, curcumin encapsulation efficiency, thermal (DSC) and rheological properties, centrifugal and freeze–thaw stability, *in vitro* digestion and colonic delivery efficiency, and fish oil oxidative stability were systematically investigated. This study aims to provide a theoretical foundation for the development of polysaccharide-based oleogel emulsions with high loading, oxidative protection, and colonic delivery.

## 2. Materials and Methods

### 2.1. Materials

Fish oil and beeswax were purchased from Skuny Bioscience Co., Ltd. (Chengdu, China) and Norevo Food Co., Ltd. (Suzhou, China), respectively. Curcumin (98%) and hyaluronic acid (97%, H874944) were purchased from Shanghai Yuanye Biotechnology Co., Ltd. (Shanghai, China) and Shanghai Maclin Biochemical Technology Co., Ltd. (Shanghai, China), respectively. Chitosan (448869), cinnamaldehyde (C108630), and coconut oil (Sunshine brand) were purchased from Sigma-Aldrich Co., Ltd. (St. Louis, MO, USA), Aladdin Reagent (Shanghai) Co., Ltd. (Shanghai, China), and Yihai Kerry Arawana Holdings Co., Ltd. (Shanghai, China), respectively. Mucin (M2378), pepsin (P7125), pancreatin (P1750), and lipase (L3126) were purchased from Sigma-Aldrich Co., Ltd. (St. Louis, MO, USA) Bile salts (B8210) were purchased from Beijing Solarbio Science & Technology Co., Ltd. (Beijing, China). Other reagents were analytical grade.

### 2.2. Preparation of the Aqueous Phase

Chitosan stock aqueous solution (20 g/kg): 2 g of chitosan was dissolved in 98 g of acetic acid solution (10 g/L) with stirring until complete dissolution, followed by overnight hydration at 4 °C. Hyaluronic acid stock aqueous solution (20 g/kg): 2 g of hyaluronic acid powder was dissolved in 98 g of deionized water until complete dissolution.

Single-polysaccharide aqueous phase: the chitosan stock solution and distilled water were mixed at a 1:1 mass ratio, and the pH was adjusted to 5.0 to obtain the single-polysaccharide (chitosan) aqueous phase.

Dual-polysaccharide aqueous phase: the chitosan stock solution, hyaluronic acid stock solution, and distilled water were mixed at a 4:1:3 mass ratio, and the pH was adjusted to 5.0 to obtain the dual-polysaccharide (containing chitosan and hyaluronic acid) aqueous phase.

### 2.3. Preparation of Emulsion Gels

Preparation of the heated oil phase: Coconut oil was heated to approximately 180 °C; beeswax and curcumin were then added. After the crystals melted, fish oil and cinnamaldehyde were added and mixed uniformly.

Preparation of the unheated oil phase: Curcumin and cinnamaldehyde were added to a mixture of coconut oil and fish oil, followed by uniform mixing with stirring.

The oil phase was poured into the aqueous phase (at a 6:4 mass ratio) and dispersed using a high-shear dispersing device (ULTRATURRAX^®^ T18, IKA, Staufen, Germany) at 8000 rpm for 3 min. The resulting emulsion was immediately cooled in an ice-water bath. During this cooling step, the beeswax and coconut oil crystallized within the oil droplets to form an in situ oleogel network, while the curcumin simultaneously underwent confined recrystallization within this structured matrix. The emulsion contained curcumin (20 g/kg), cinnamaldehyde (10 g/kg), fish oil (200 g/kg), and beeswax at 0% (0 g/kg), 1% (10 g/kg), 2% (20 g/kg), or 6% (60 g/kg). The above percentages represent mass fractions.

Emulsions with single chitosan as the aqueous phase, an unheated internal oil phase, and no beeswax were denoted as CS-No heating; those with single chitosan as the aqueous phase, a heated internal oil phase, and no beeswax were denoted as CS; those with chitosan/hyaluronic acid dual polysaccharides as the aqueous phase, a heated internal oil phase, and no beeswax were denoted as CS-HA; those with chitosan/hyaluronic acid dual polysaccharides as the aqueous phase, a heated internal oil phase, and 10 g/kg beeswax were denoted as CS-HA + 1%; those with chitosan/hyaluronic acid dual polysaccharides as the aqueous phase, a heated internal oil phase, and 20 g/kg beeswax were denoted as CS-HA + 2%; and those with chitosan/hyaluronic acid dual polysaccharides as the aqueous phase, a heated internal oil phase, and 60 g/kg beeswax were denoted as CS-HA + 6%. The percentage symbols in CS-HA + 1%, CS-HA + 2%, and CS-HA + 6% are retained only as treatment codes; the actual beeswax concentrations are 10, 20, and 60 g/kg, respectively. In this study, chitosan (CS) and hyaluronic acid (HA) served as natural polymeric emulsifiers and stabilizers, with cinnamaldehyde acting as an interfacial cross-linker.

### 2.4. Determination of Curcumin Encapsulation Efficiency

After the emulsion samples were centrifuged at 4000 rpm for 2 h, the upper free oil and the precipitated curcumin crystals were removed. The emulsion layer was collected and extracted with chloroform, and the curcumin content was then determined using a UV–Vis spectrophotometer at 420 nm.

Encapsulation efficiency (EE) (%) = (Cx/C0) × 100% (1)

where Cx is the curcumin content in the emulsion layer after centrifugation and C0 is the total curcumin content before centrifugation.

### 2.5. Particle Size Measurements

The particle size distribution of the emulsions was determined using a Malvern Mastersizer 3000 (Malvern Instruments Ltd., Malvern, UK). The emulsion was gradually added dropwise to the measurement chamber until the shading was between 8% and 15%. Each sample was measured three times. The instrument software provides the surface area-based mean particle size (D_3,2_) and the volume-based mean particle size (D_4,3_).

### 2.6. Emulsion Morphology

#### 2.6.1. Optical Microscopy

Optical microscopy (Dmi8, Leica Microsystems CMS GmbH, Wetzlar, Germany) at 100× or 400× magnification was employed to characterize the morphology of the emulsions. For sample preparation, approximately 10 μL of the emulsion was applied onto a glass slide, and a coverslip was gently placed over the sample before observation. Digital images were recorded using the camera (Leica K3C).

#### 2.6.2. Cryo-Scanning Electron Microscopy

The microstructure of the emulsions was observed using a cryo-scanning electron microscope (Cryo-SEM, Regulus 8100, HITACHI, Tokyo, Japan). Samples were placed on a sample holder and rapidly plunged into liquid nitrogen slush for ultra-rapid freezing fixation. The frozen samples were transferred under vacuum to the preparation chamber, fractured at −140 °C, sublimated at −80 °C for 20 min to remove surface ice crystals, and then sputter-coated with a platinum alloy for 60 s. The prepared samples were imaged at −140 °C under an accelerating voltage of 1.0 kV.

### 2.7. DSC Measurement

DSC analysis was performed using a differential scanning calorimeter (DSC200, Hitachi, Tokyo, Japan). The instrument was maintained and periodically calibrated according to the manufacturer’s standard procedure. Based on a previous method with slight modifications [23], 5–7 mg of emulsion sample was weighed into an aluminum crucible and sealed by pressing, with an empty sealed aluminum crucible serving as the reference. The sample was heated from 25 °C to 220 °C at a rate of 10 °C/min under a nitrogen atmosphere at a flow rate of 30 mL/min. A single heating scan was performed for each sample, and the DSC curve as a function of temperature was obtained.

### 2.8. Rheological Properties

Based on previous methods [24], the rheological properties of the emulsions were measured using a rheometer (MCR302, Anton Paar GmbH, Graz, Austria). Shear rate sweeps were performed to obtain viscosity–shear rate curves (0.1–100 s^−1^); strain sweeps (0.01–1000%) were conducted to determine the linear viscoelastic region and yield behavior; and frequency sweeps (1–100 rad/s) were carried out to examine the variations in storage modulus (G′) and loss modulus (G″) with angular frequency, with the shear strain fixed at 0.1% for the frequency sweeps. The shear stress (τ) was calculated from the measured apparent viscosity and shear rate (τ = η × γ), and the resulting τ–γ data were fitted to the Power Law model (τ = Kγ^n^) using Origin software (Version 2018) to obtain the consistency index (K), flow behavior index (n), and coefficient of determination (R^2^).

### 2.9. Centrifugal Stability

Based on a previous study [25], the transmission profiles of the emulsions were obtained using a LUMi-Sizer stability analyzer (LUMiSizer^®^, LUM GmbH, Berlin, Germany), and the instability index was determined via the instrument software. Test conditions: 2000 rpm, 25 °C, and 2 h.

### 2.10. Freeze–Thaw Stability

Based on previous methods with appropriate modifications [26], all emulsion samples were stored in a −20 °C freezer for 22 h, then removed and thawed at 25 °C for 2 h, which constituted one freeze–thaw cycle. The emulsions were subjected to three freeze–thaw cycles, and photographs of the emulsion appearance were taken after each cycle. After three freeze–thaw cycles, the emulsions were examined by electron microscopy and subjected to stability testing (2000 rpm, 1 h) to obtain the curve of instability index versus time.

### 2.11. In Vitro Digestion

The *in vitro* digestion simulation protocol (the INFOGEST static) was based on previous studies, employing a three-stage *in vitro* digestion model mimicking the oral–gastric–intestinal tract to conduct continuous digestion of the emulsion [27]. Simulated saliva (SSF), gastric (SGF), and intestinal fluids (SIF) were prepared according to established methods and maintained at 37 °C throughout the experiment [28]. The activities of the enzymes were provided by the supplier’s certificate of analysis and were used to prepare the digestion fluids at the final activities recommended by INFOGEST. The final activities in the digestion mixtures were mucin 75 U/mL, pepsin 2000 U/mL, pancreatin 100 U/mL, lipase 60 U/mL, and bile salts were used at 10 mM.

Oral Phase: A defined amount of sample was diluted with water to a final volume of 5 mL. A 4 mL volume of SSF containing activated mucin was added, and the pH was adjusted to 6.8. Then, 25 µL of CaCl_2_·2H_2_O solution (0.3 M) and 0.975 mL of water were added. The mixture was incubated at 37 °C with oscillation at 100 rpm for 10 min.

Gastric Phase: Following oral digestion, 8 mL of SGF containing activated pepsin was added, together with 5 µL of CaCl_2_·2H_2_O solution (0.3 M) and 1.995 mL of water. The pH was adjusted to 3.0, and the mixture was incubated at 37 °C with oscillation at 100 rpm for 2 h.

Intestinal Phase: After gastric digestion, 7.5 mL of SIF was added, and the pH was adjusted to 6.5. Subsequently, 3.5 mL of activated bile salts, 2.5 mL of pancreatin solution, and 2.5 mL of lipase solution (all dissolved in SIF) were added, along with 40 µL of CaCl_2_·2H_2_O solution (0.3 M) and 3.96 mL of water. The pH was adjusted to 7.0, and the mixture was incubated in a 37 °C water bath with shaking for 2 h, with pH readjustments to 7.0 at 5, 15, 30, 60, and 90 min.

Stability, bioaccessibility and colon delivery efficiency of curcumin: The free oil group, prepared by directly suspending curcumin in the oil phase without beeswax and emulsification, was used as the control for the *in vitro* digestion evaluation. All emulsion groups were compared with this control. After intestinal digestion, the samples were immediately placed in an ice-water bath for 10 min to terminate the digestion process. The digestates were then centrifuged at 15,000 rpm for 30 min, and the middle layer was collected as the micellar layer. Curcumin in both the total digestate and the micellar phase was extracted using ethanol for demulsification, followed by chloroform extraction. The absorbance of curcumin was measured at 420 nm using a UV–Vis spectrophotometer, and its concentration was calculated based on a standard curve.

Stability (%) = C_d_/C_0_ × 100% (2)

Bioaccessibility (%) = C_m_/C_d_ × 100% (3)

Estimated colon delivery efficiency (%) = (C_d_ − C_m_)/C_0_ × 100% (4)

C_d_: Curcumin concentration in the digesta after the intestinal digestion phase. C_0_: Initial concentration of curcumin added to the simulated digestive system. C_m_: Curcumin concentration in the micellar layer. Estimated colon delivery efficiency refers to the proportion of curcumin delivered to the colon stage. The colonic phase was not included in this protocol. The “estimated colon delivery efficiency” therefore represents the fraction of curcumin retained in the digestate after small intestinal digestion and not incorporated into mixed micelles, and potentially available for colonic delivery, rather than a directly measured colonic release.

### 2.12. Oxidative Stability Assessment

After preparation, the oil phase and freshly prepared emulsions (without curcumin) were placed in a 45 °C oven for accelerated storage, and the contents of lipid oxidation products were determined on days 0, 7, and 14.

The method for determining the primary oxidation products (POV) was as follows [29]: In brief, 300 mg of sample was combined with 1.5 mL of isooctane/isopropanol (3:1, *v*/*v*). The mixture was vortexed for 5 min and then centrifuged at 5000 rpm for 2 min. A 200-μL aliquot of the upper phase was mixed with 2.8 mL of methanol/butanol (2:1, *v*/*v*). Thereafter, 15 μL of NH_4_SCN (3.94 mol/L) and 15 μL of Fe^2+^ solution were added. The Fe^2+^ solution was prepared by dissolving 0.806 g of BaCl_2_·2H_2_O in 25 mL of water and 1 g of FeSO_4_·7H_2_O in 25 mL of water; after dissolution, the two solutions were combined, 1 mL of 10 M HCl was added, and the mixture was centrifuged to obtain the supernatant. The reaction was conducted in the dark for 20 min. Absorbance was measured at 510 nm with a microplate reader (FlexA-200, Hangzhou Allsheng Instruments Co., Ltd., Hangzhou, China), and the hydroperoxide concentration was calculated using a hydrogen peroxide standard curve.

Secondary oxidation products (MDA) were determined using a malondialdehyde (MDA) assay kit (Nanjing Jiancheng Bioengineering Institute, Nanjing, China) according to the manufacturer’s instructions.

### 2.13. Statistical Analysis

All experimental measurements were performed in triplicate. The data were analyzed by one-way analysis of variance (ANOVA) using SPSS 26.0, and Tukey’s test was used to determine significant differences between means, with the significance level set at *p* < 0.05.

## 3. Results and Discussion

### 3.1. Physicochemical Properties of High-Load Emulsion Gels

**Figure 1:**
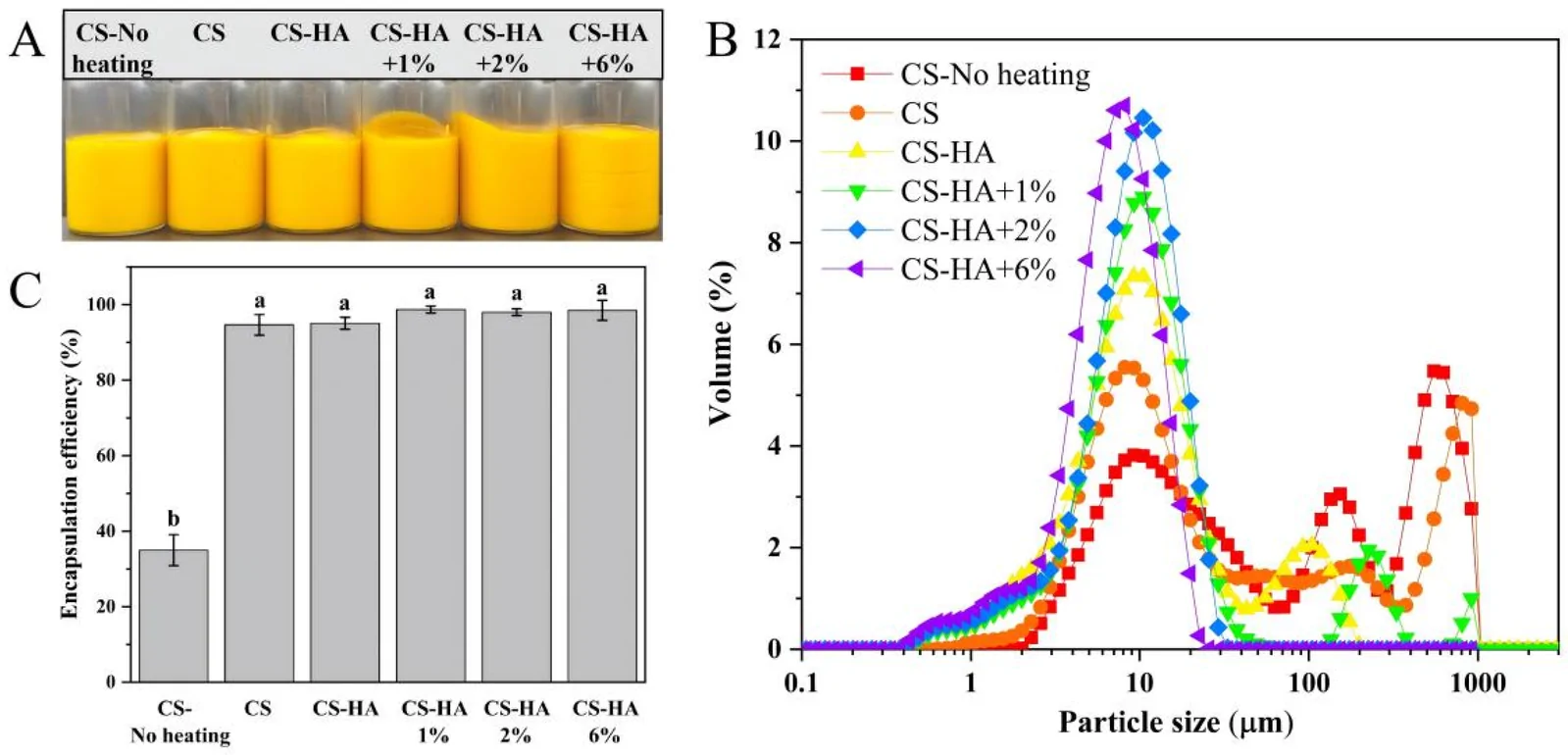
Appearance of the emulsion (**A**); particle size distribution of the emulsion (**B**); curcumin encapsulation efficiency (**C**). Different lowercase letters (a, b) in (**C**) indicate significant differences (*p* < 0.05).

The freshly prepared emulsions containing curcumin and fish oil were uniformly yellow, with no oiling off or phase separation (Figure 1A). As shown in Figure 1B, the particle size distribution varied significantly with the interfacial composition and beeswax concentration. Except for CS-HA + 2% and CS-HA + 6%, all emulsions exhibited multimodal distributions, displaying a primary population near 10 μm and a secondary population of several hundred micrometers. This secondary peak is attributed to the flocculation and/or coalescence of small oil droplets into large aggregates. In contrast, CS-HA + 2% and CS-HA + 6% showed unimodal distributions centered near 10 μm. The transition from a multimodal to a unimodal distribution is driven by the internal gel strength. At lower beeswax concentrations (0% and 1%), the oil-phase gel network was insufficient to fully suppress the growth of curcumin crystals after heating and cooling. These large crystals likely disrupted the oil–water interface, inducing droplet coalescence. Conversely, the higher beeswax concentration (2% and 6%) increased the gel strength, which strongly restricted crystal growth and prevented interface disruption, thus eliminating large aggregates.

This structural transition is clearly reflected in the mean particle sizes (Table 1). The D_3,2_ values (surface-area-weighted) and D_4,3_ values (volume-weighted) differ by more than an order of magnitude for multimodal samples (e.g., D_3,2_ = 17.73 μm vs. D_4,3_ = 215.67 μm for CS-No heating), because D_4,3_ is highly sensitive to a few large aggregates. By contrast, for the uniform CS-HA + 2% and CS-HA + 6% emulsions, the D_4,3_ values (10.07 and 7.71 μm) are much closer to their D_3,2_ values (5.63 and 4.57 μm), confirming the absence of large aggregates. Importantly, the statistical analysis in Table 1 confirms that the particle sizes (both D_3,2_ and D_4,3_) varied significantly among the different emulsion formulations (*p* < 0.05), as indicated by the different superscript letters.

The encapsulation efficiency of curcumin in the emulsion prepared with an unheated oil phase (~35%) was significantly lower than that in heat-prepared emulsions (all >95%, Figure 1C). This difference is fundamentally governed by the lipophilic and thermal properties of curcumin. Curcumin is highly hydrophobic but has limited solubility in liquid oil at room temperature (only a few mg/g), and its high melting point (~183 °C) means it exists as solid crystals under ambient conditions. Because the loading concentration in this study (20 mg/g) far exceeded its solubility limit, heating was required to completely melt the curcumin crystals. Upon emulsification and subsequent cooling, curcumin recrystallized within the confined space of the oil droplets. This spatial confinement (provided primarily by the oil–water interface, and further reinforced by the CS/HA layer and beeswax network) restricted the crystals to a fine size, preventing them from growing into large crystals that would otherwise disrupt the interfacial film and cause coalescence. Similarly, interfacial composition and structure have been shown to regulate the crystallinity and storage stability of β-carotene in lipid dispersion systems [30]. The confinement effect of emulsion droplets can itself alter lipid nucleation and crystal morphology, with smaller droplets leading to smaller and more irregular crystals [31,32]. In contrast, without the heating–cooling treatment, large native curcumin crystals remained in the oil phase. These large crystals disrupted the oil–water interface, leading to droplet aggregation and sedimentation during centrifugation, which explains the low EE (~35%). Therefore, the heating–cooling process was treated as a fundamental prerequisite for constructing this high-load system, and subsequent optimizations (CS-HA and beeswax addition) were evaluated under this heated condition.

**Figure 2:**
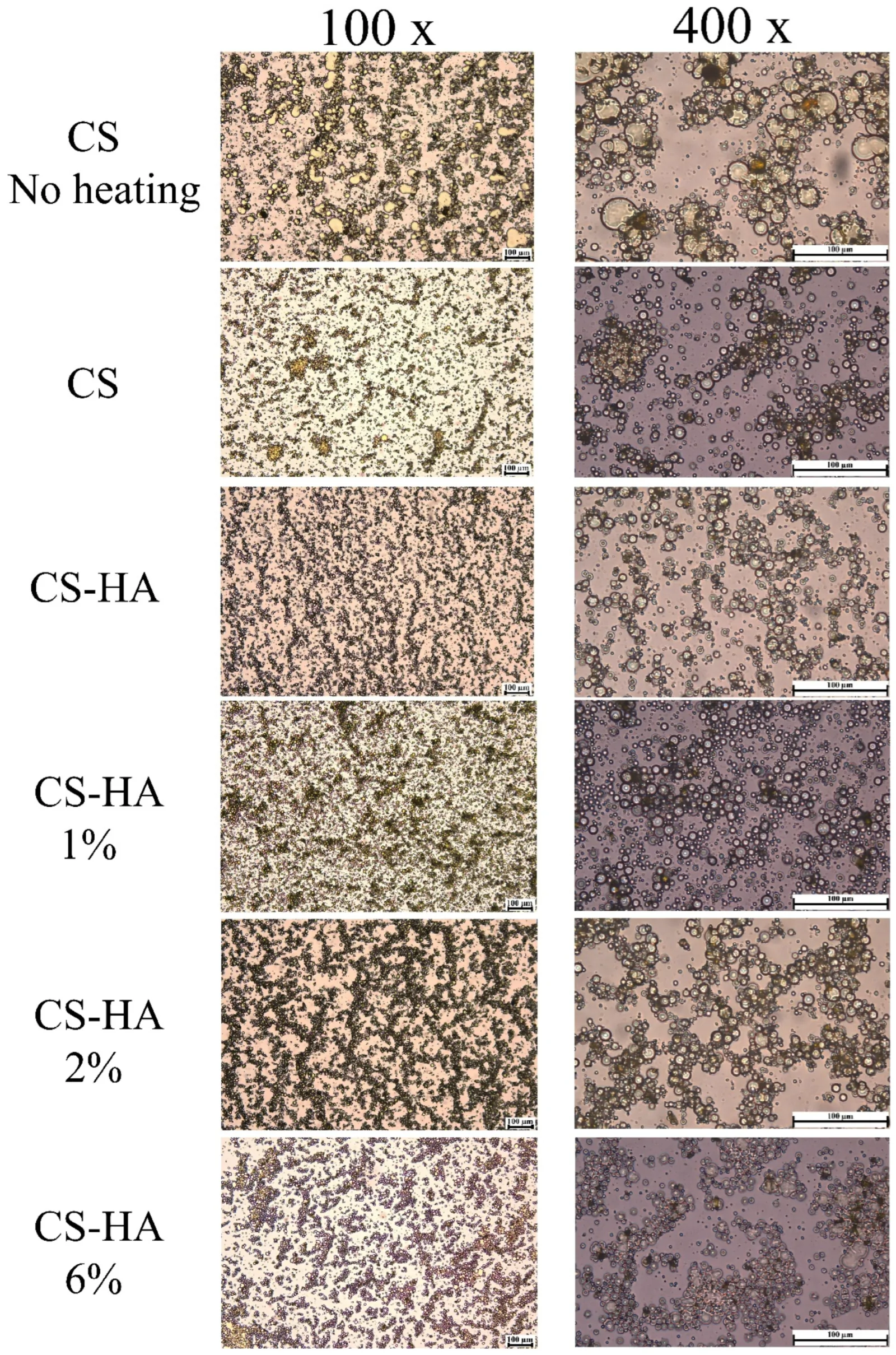
Microscopic morphology of the curcumin-loaded emulsion (at 100× and 400× magnification) (white scale bar is 100 µm).

The microscopic morphology of curcumin-loaded emulsions prepared under different conditions and with different beeswax concentrations was shown in Figure 2. At 100× and 400× magnification, all samples formed spherical oil droplets, consistent with typical oil-in-water emulsions. This spherical droplet morphology is in agreement with previous optical microscopy observations of curcumin-loaded O/W emulsions [33]. However, droplet morphology and dispersion differed markedly among groups. The chitosan-stabilized emulsion with unheated oil phase (CS-No heating) exhibited relatively large droplets with evident local coalescence; some droplets showed irregular shapes and rough contours. Heat-treated CS emulsions showed an overall reduction in droplet size but remained dominated by clusters of numerous small droplets. In contrast, the dual-polysaccharide system incorporating hyaluronic acid (CS-HA) displayed significantly smaller and more uniform droplets. Among these, CS-HA emulsions containing 2% and 6% beeswax had the smallest and most uniformly dispersed droplets, with almost no large aggregates.

The observed microscopic morphological differences highlight the synergistic regulation of crystallization by droplet confinement and interfacial barrier. In the control group, the oil phase was not heated, resulting in large native curcumin crystals dispersed in the continuous oil phase at high loading. During emulsification, these large crystals easily disrupted the oil–water interface film, inducing droplet coalescence. Consequently, the resulting emulsion droplets were larger and more polydisperse. In contrast, heating the oil phase promoted thorough melting and re-dissolution of curcumin with the beeswax. Upon emulsification followed by rapid cooling, curcumin underwent re-crystallization within the pre-formed dispersed droplets. Due to the confinement imposed by the interface film, the limited growth space restricted the crystals to a fine size, preventing the destructive penetration of large crystals across the interface. This yielded a more uniform and stable emulsion. The addition of beeswax further regulated this crystallization process: the beeswax crystals could initially form a gel network scaffold in the oil phase, thereby locking the curcumin crystals within the oil gel phase and inhibiting their migration and breakthrough of the oil–water interface. As the beeswax concentration increased, the confinement and encapsulation effects on the crystals became stronger, leading to even more uniform droplets. At the interfacial level, hyaluronic acid and chitosan formed a dense polysaccharide barrier through electrostatic assembly and their mutual interactions. This interfacial film may possess higher mechanical strength than that formed by chitosan alone, thereby enhancing the overall emulsion stability while also preventing contact and fusion among crystals from adjacent droplets, effectively blocking their growth. Therefore, the combination of heating-induced droplet-confined re-crystallization, beeswax-regulated oil–gel structure, and the dual-polysaccharide interface constitutes the key to achieving a high-load curcumin emulsion with uniform particle size and high dispersion stability.

**Figure 3:**
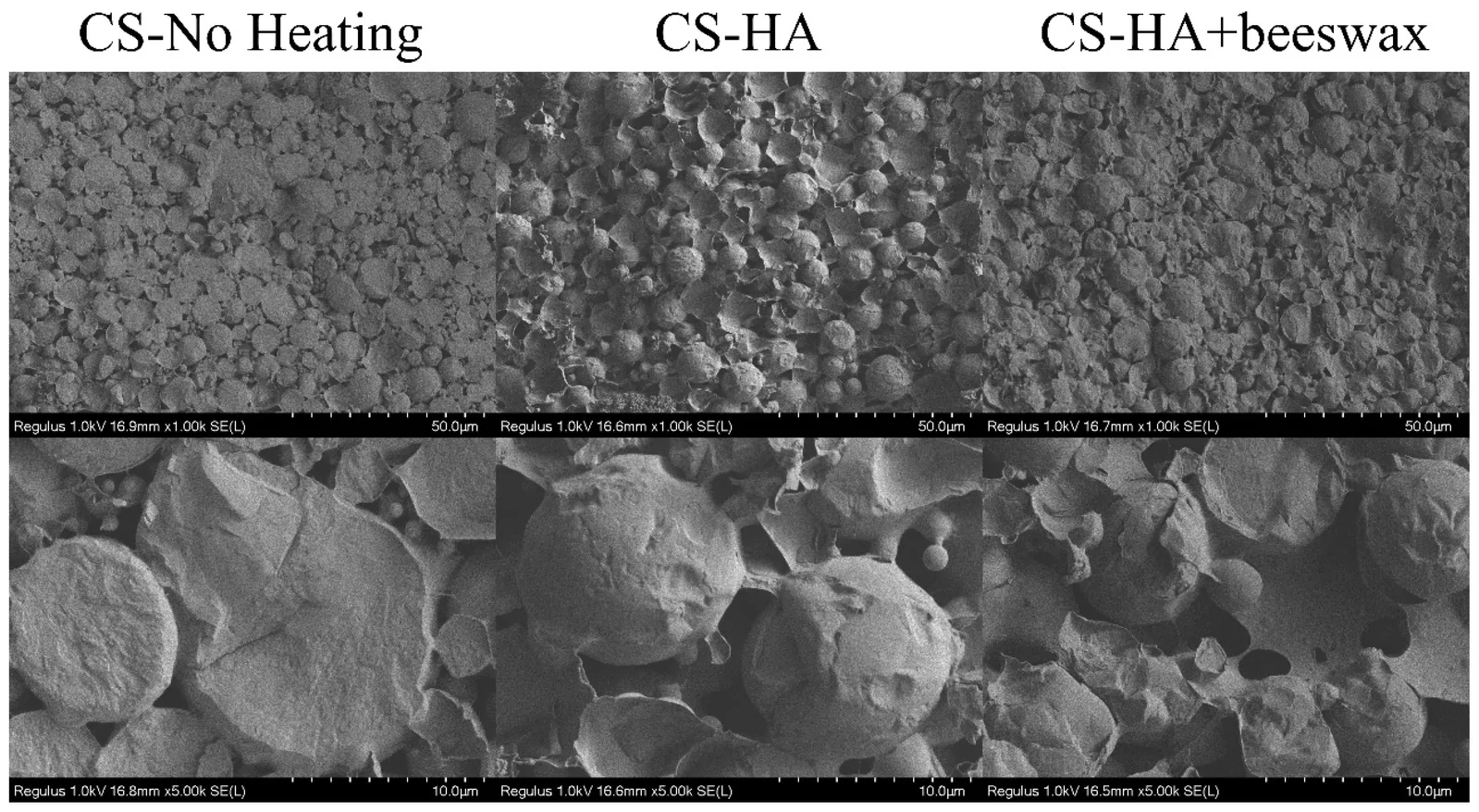
Microstructure of the emulsion: cryo-scanning electron microscope (cryo-SEM).

Cryo-SEM further reveals the microscopic structural differences in the emulsions under varying interfacial compositions (Figure 3). In the control group (CS-No heating), the particles remained spherical with smooth surfaces, and the particles were independent with no obvious interfacial adhesion; however, the presence of large irregular oil droplets indicates oil-droplet aggregation. In the dual-polysaccharide system (CS-HA), a continuous wrinkled membrane-like matrix appeared between particles, the particle boundaries became blurred, and the particles were enveloped by the polysaccharide matrix. With the addition of beeswax (CS-HA + beeswax), the sphericity of the particles further decreased, large wrinkled membrane-like structures were observed on the particle surfaces, and a continuous membrane layer structure with pores emerged. The emulsion loaded with luteolin, stabilized by chitosan and whey protein isolate, also exhibited a three-dimensional network wrapping around the oil-droplet surface [34].

The observed morphological changes can be interpreted from two aspects: the interfacial film and the crystallization of the oil phase. The single chitosan interface in the control group had limited strength and a relatively thin film, resulting in smooth, independent spherical particles. Hyaluronic acid, electrostatically assembled onto the chitosan interface, formed a dense polysaccharide barrier. This thickened and interconnected interfacial film manifested as a continuous wrinkled membrane-like matrix in the particle gaps, confirming that the dual-polysaccharide interface possesses higher mechanical strength and structural continuity. Upon addition of beeswax, the oil phase underwent crystallization to form an oleogel network during heating–cooling, synergizing with the polysaccharide interface to drive the system toward gelation. During the freeze-sampling process, water in the aqueous phase was less prone to volatilization, and the confinement of the continuous phase and interfacial film induced partial droplet collapse and shrinkage, further highlighting the shaping effect of the gel network on droplet morphology. Collectively, these results demonstrate that the synergy between the dual-polysaccharide interface and the beeswax oleogel network significantly altered the interfacial structure and gelation degree of the emulsion, providing a structural basis for the stabilization of high-load curcumin emulsions.

### 3.2. Thermal Analysis (DSC) of High-Load Emulsion Gels

**Figure 4:**
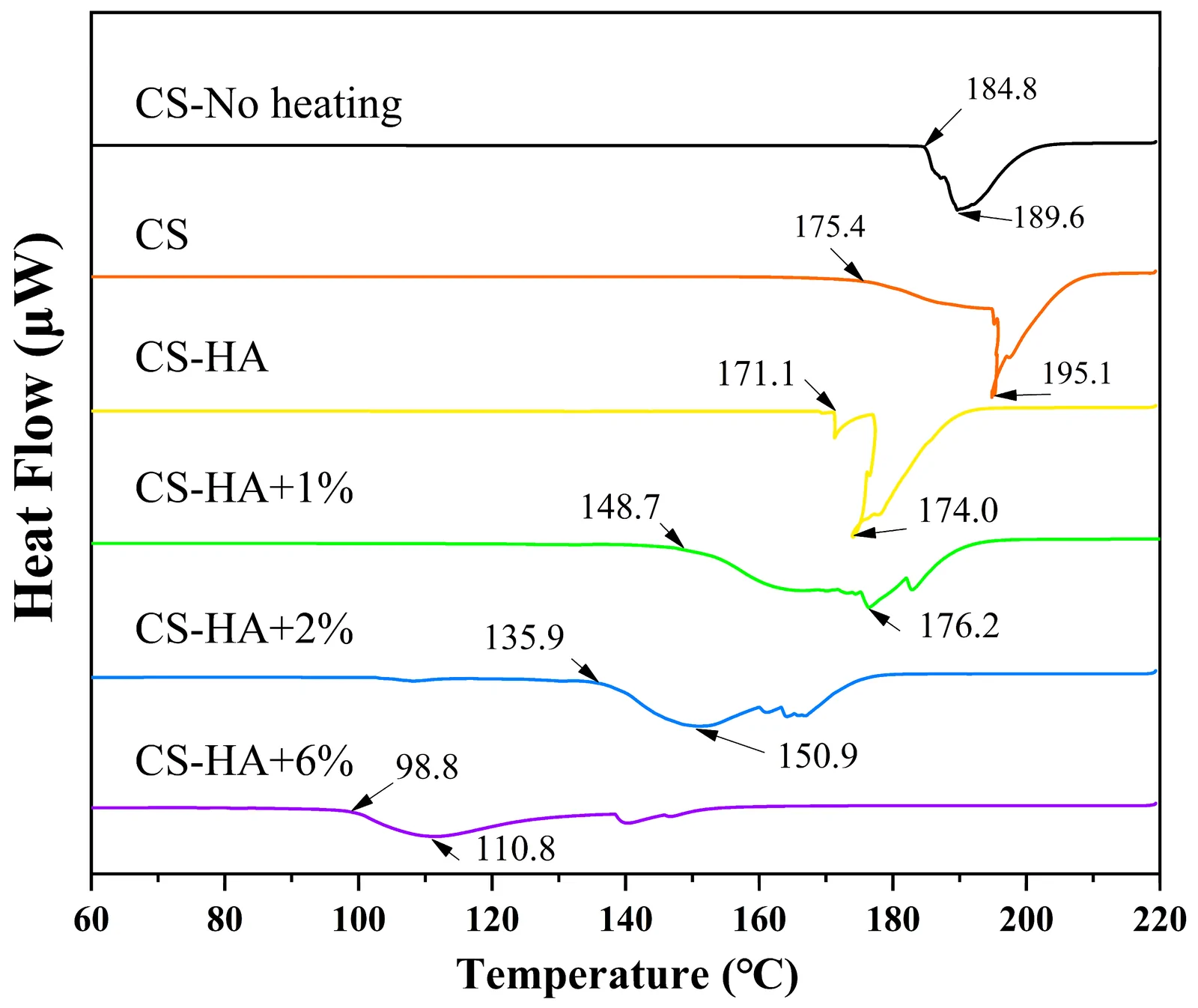
DSC curves of emulsions loaded with curcumin (downward peaks indicate endothermic direction; heat flow in μW). For clarity, the DSC curves are vertically offset.

DSC thermal analysis curves of different curcumin-loaded emulsions were shown in Figure 4. For all samples, an endothermic peak was observed during heating, corresponding to the melting of components (mainly curcumin crystals) in the emulsions. It should be emphasized that this DSC analysis focuses on the confined recrystallization behavior of curcumin within the emulsion structure, revealing how spatial confinement and the oleogel network alter the thermodynamic properties of the crystals. The onset temperature and peak temperature of the endothermic peaks varied significantly among the groups. For the control group (CS-No heating), the onset temperature of the endothermic peak was 184.8 °C and the peak temperature was 189.6 °C, consistent with the melting point of native curcumin crystals (~183 °C), with a sharp peak shape. For the heated single-chitosan emulsion (CS), the onset temperature decreased to 175.4 °C and the peak shape broadened with a peak temperature of 195.1 °C, likely due to an asymmetric melting curve and partial crystal aggregation under the single-layer interface. For the dual-polysaccharide system (CS-HA), the onset temperature further decreased to 171.1 °C and the peak temperature was 174.0 °C. Upon introduction of beeswax, the onset and peak temperatures continuously shifted to lower temperatures with increasing beeswax concentration. The onset temperatures of CS-HA + 1%, +2%, and +6% were 148.7 °C, 135.9 °C, and 98.8 °C, respectively, and the peak temperatures were 176.2 °C, 150.9 °C, and 110.8 °C, respectively, with the peak shape gradually changing from sharp to broad and flattened.

The evolution of the endothermic peak onset temperature and peak shape can be explained by curcumin crystal size and oleogelation. In the control group, without heating, large native curcumin crystals coexisted in the oil phase with high crystallinity, so melting occurred in a concentrated manner near their intrinsic melting point, resulting in the highest onset temperature and sharpest peak shape. Upon heating, curcumin was re-dissolved and underwent confined re-crystallization within the droplets. This spatial confinement led to smaller crystal sizes and increased lattice defects, raising the surface energy and thus lowering the melting onset temperature. This is consistent with the microscopic structure described above, where the heating groups showed more uniform droplets and crystals confined by the droplets. Yu et al. also showed that the DSC peak shape and melting temperature of solid lipid nanoparticles containing curcumin are closely related to the crystal structure and crystallization morphology [35]. The introduction of hyaluronic acid to construct the dual-polysaccharide interface enhanced the interfacial film strength and barrier properties, further restricting crystal growth space. This resulted in smaller curcumin crystals, leading to a further reduction in the onset temperature. The addition of beeswax significantly altered the thermal behavior of the oil phase. Since the melting of neat beeswax is ~62 °C, well below the 98.8–189.6 °C window observed here, the endothermic peaks in this study cannot be attributed to independent melting of beeswax itself. Instead, the crystallization of beeswax with curcumin, fish oil, and coconut oil formed an oleogel network in which curcumin crystals were dispersed and “anchored” within the oleogel. This interaction involved eutectic crystallization or partial miscibility with lipid components such as beeswax, disrupting the crystal regularity. Consequently, the melting peak transitioned from sharp to broad and flattened, with the onset temperature decreasing markedly. A higher beeswax concentration produced a denser network, exerting stronger suppression on crystal size and ordering, reaching an onset temperature of 98.8 °C at 6% beeswax. Sarkisyan et al. quantitatively investigated the relationship between the yield strength of beeswax oil gels and the crystal size distribution, providing a theoretical basis for the correlation that a denser gel network corresponds to smaller crystal sizes [36]. Li et al. demonstrated that the coexistence of liquid oil and oleogelators in oleogels increases the free energy barrier for the crystallization of guest molecules (cocoa butter), thereby inhibiting guest crystal growth and polymorphic transitions [37]. The DSC results are corroborated by the particle size and microscopic morphology observations, collectively indicating that confined recrystallization, barrier effects of the dual-polysaccharide interface, and regulation by the beeswax oleogel synergistically reduce curcumin crystal sizes and lower their melting point.

### 3.3. Rheological Properties of the Emulsion Gels

**Figure 5:**
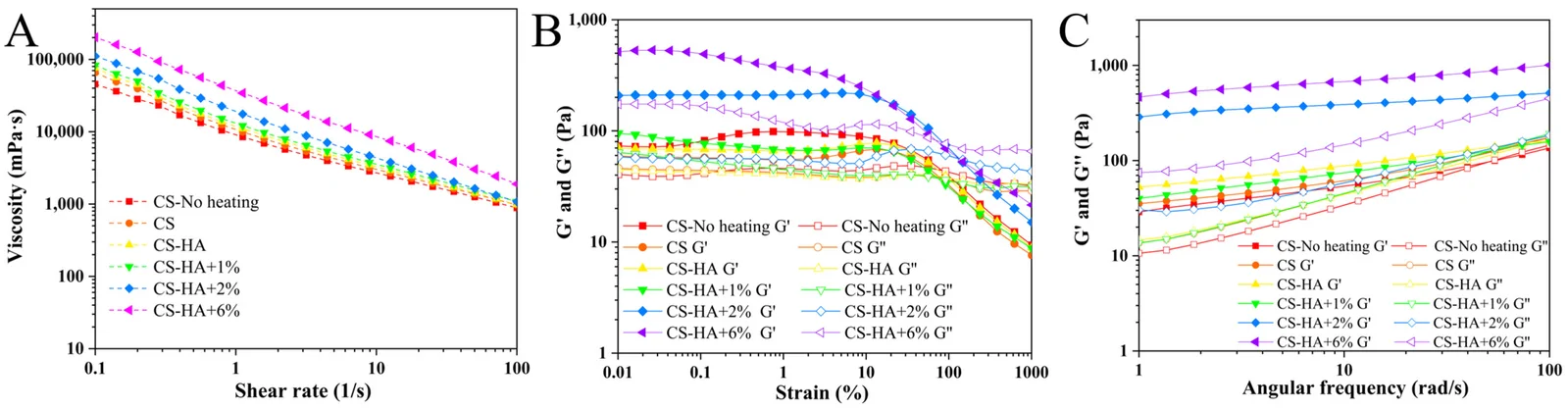
The shear rate–viscosity curve (**A**); the storage modulus and loss modulus (G′ and G′) vary with strain (**B**); the storage modulus and loss modulus (G′ and G′) vary with angular frequency (**C**).

Figure 5 presents the rheological behavior of emulsions with different aqueous and oil phase compositions. The shear viscosity curves (Figure 5A) show that all emulsions exhibit typical shear-thinning behavior, with viscosity decreasing as the shear rate increases. The groups exhibited significant differences in viscosity, with the overall order: CS-HA + 6% > CS-HA + 2% > CS-HA + 1% > CS-HA > CS > CS-No heating. The introduction of hyaluronic acid and the increase in beeswax concentration both increased the system viscosity. To further elucidate the flow behavior of the emulsions, the shear stress–shear rate data were fitted using the Power Law model (τ = K$\dot{\gamma }$^n^), and the fitting results are listed in Table 2. The R^2^ values of all samples were above 0.996, indicating that the Power Law model accurately describes the flow characteristics of the emulsion system. The consistency index K reflects the flow resistance or apparent viscosity level of the fluid at unit shear rate; a larger K value indicates higher structural consistency of the system. As shown in Table 2, K increased from 9.574 Pa·s^n^ for CS-No heating to 38.128 Pa·s^n^ for CS-HA + 6% and showed an increasing trend with the introduction of HA and increasing beeswax concentration. The flow behavior index n characterizes the degree to which the fluid deviates from Newtonian behavior. The n values of all samples were well below 1 (0.196–0.325), confirming that these emulsions are typical pseudoplastic fluids [38]. The fitted rheological parameters above provide quantitative support for the qualitative ranking of apparent viscosity in Figure 5A. Strain sweeps (Figure 5B) revealed that, at low strains, G′ > G″ for all samples, indicating gel-like behavior. As strain increased, a transition from solid-like to liquid-like occurred after the critical yield point in all systems. The CS-HA + 6% and CS-HA + 2% systems displayed the widest linear viscoelastic regions and the highest yield strains (approximately 100%), while the low-concentration and pure chitosan emulsions showed lower yield strains, followed by a rapid decline in modulus post-yield. Frequency sweeps (Figure 5C) demonstrated that all samples maintained G′ > G″ across the entire frequency range, with G′ remaining nearly constant (platform-like) as frequency increased, whereas G″ increased more noticeably. This indicates a stable elastic network in which viscous contributions are more sensitive to frequency. The modulus ranking was consistent with the linear region in the strain sweeps, with higher beeswax concentrations yielding higher moduli.

These rheological features reveal the synergistic construction of a gel network by oleogel and dual-polysaccharide interfaces. All samples exhibited shear-thinning behavior and G′ > G″, indicating the formation of a three-dimensional network with solid-like characteristics [39]. Comparison among groups showed that the single-chitosan systems had the lowest viscosity and modulus, with the unheated group being the lowest, indicating a weak interfacial film and insufficient network crosslinking. Upon introducing hyaluronic acid, HA electrostatically assembled onto the chitosan interface, enhancing interfacial film strength and promoting inter-droplet interactions, thereby increasing system viscosity and modulus. Beeswax played a dominant role: its crystallization constructed a gel skeleton in the oil phase and anchored curcumin crystals, fish oil, and coconut oil within the oleogel network, forming a continuous crosslinked structure within and between dispersed droplets. Higher beeswax concentration led to a denser gel network, thus increasing viscosity, G′, G″, and yield strain [40]. CS-HA + 6% exhibited the highest viscosity and modulus and the widest yield strain, indicating that it formed the toughest and most deformation-resistant gel network. This rheological trend corroborates the previous microstructural and DSC results: heat-confined recrystallization, beeswax oleogelation, and the dual-polysaccharide interface act synergistically to collectively determine the degree of gelation and mechanical strength of the emulsions. A moderate gel network not only helps inhibit the sedimentation and migration of curcumin crystals and maintain the long-term stability of high-load emulsions, but also provides the structural integrity required for colon-targeted delivery.

### 3.4. Stability of the Emulsions

(1) Centrifugal stability

**Figure 6:**
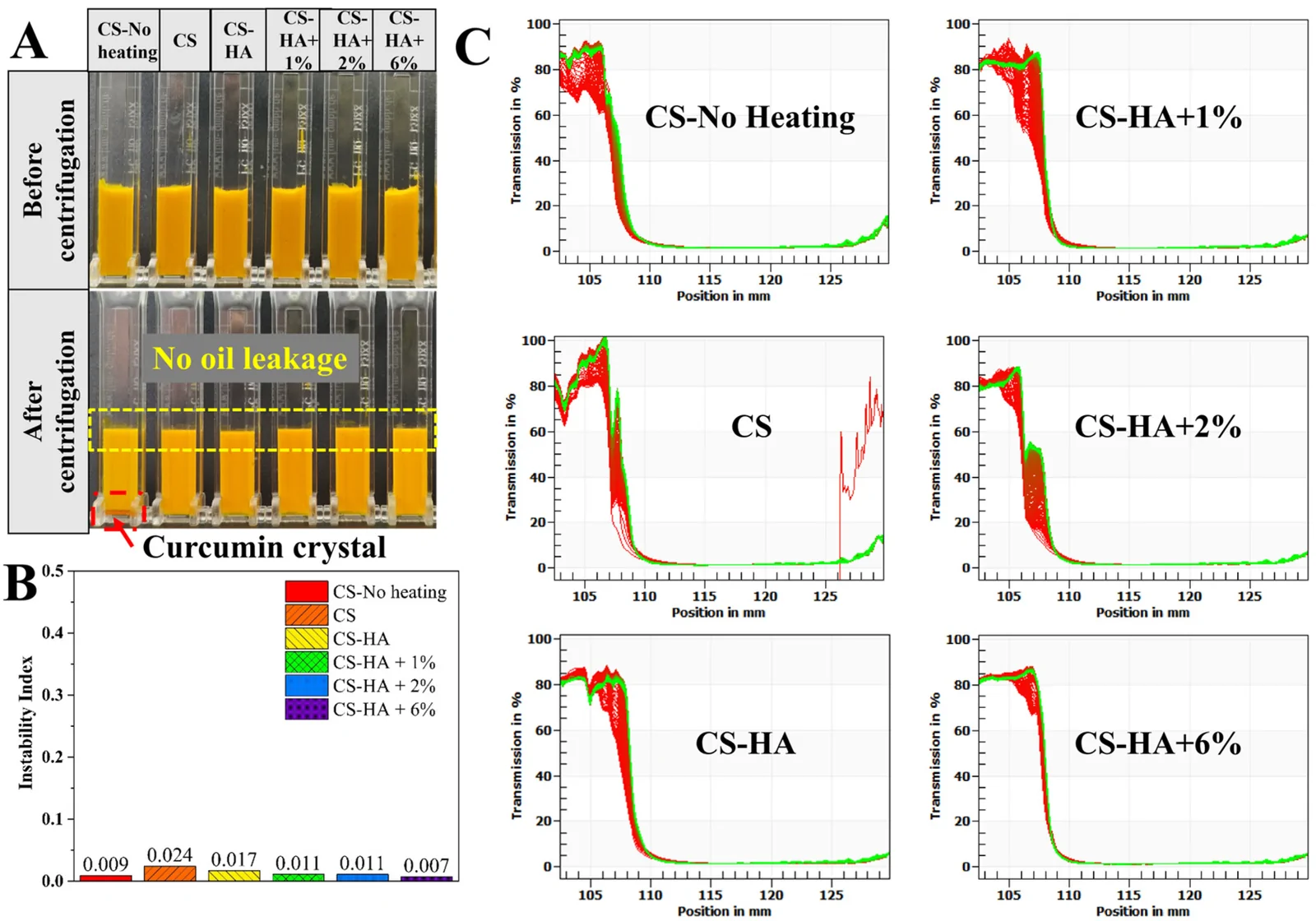
Centrifugal stability of the emulsions. Appearance of the emulsions before and after centrifugation (**A**); Instability index of the emulsions (**B**); Transmission profiles of different emulsions during centrifugation (**C**).

The centrifugal stability of emulsions with different compositions was evaluated (Figure 6). As shown in the visual appearance (Figure 6A), all samples exhibited a homogeneous bright-yellow appearance without phase separation before centrifugation. After centrifugation for 2 h, no oil leakage was observed in any group. However, curcumin crystals were deposited at the bottom of the tube in the CS-No heating group (red arrow), whereas no visible crystal precipitation was detected in the other groups. The instability indices (Figure 6B) were all well below 0.03, indicating generally good centrifugation stability. The transmittance profiles (Figure 6C) showed only minor shifts during centrifugation, with limited differences between the pre- and post-centrifugation curves, suggesting no pronounced oil–water phase separation. Notably, the red and green curves of the CS-HA + 6% group were nearly superimposed, indicating the highest centrifugation stability.

The differences in centrifugation stability can be attributed primarily to the strength of the interfacial film and the degree of oil-phase gelation. The consistently low instability indices (<0.03) and absence of oil leakage indicate that all emulsions exhibited strong resistance to centrifugation-induced phase separation. Although the CS-No heating group had a relatively low instability index (0.009), distinct curcumin crystal deposition was observed at the bottom of the tube, suggesting that, in the absence of heating, curcumin remained predominantly in the form of relatively large native crystals that were difficult to effectively retain within the interfacial layer and were therefore prone to sedimentation under centrifugal forces. The CS group exhibited the highest instability index, indicating that the interfacial film formed solely by chitosan provided limited mechanical strength, allowing greater droplet migration under the centrifugal field. Incorporation of hyaluronic acid to form a dual-polysaccharide interfacial layer enhanced interfacial film strength and improved droplet–droplet interactions and network stability, thereby markedly reducing the instability index and eliminating visible crystal sedimentation. The addition of beeswax further enhanced centrifugation stability. Crystallization of beeswax within the oil phase generated a gel-like network that effectively immobilized curcumin crystals within the droplets, thereby suppressing their sedimentation and migration under centrifugal forces. As the beeswax concentration increased, the resulting gel network became progressively denser, leading to greater resistance to centrifugation-induced destabilization [41]. The transmittance curves of the CS-HA + 6% group during centrifugation almost overlapped, with the lowest index, indicating the most stable structure. The centrifugal stability results corroborate the micromorphological, DSC, and rheological results, demonstrating that the synergy of heat-confined recrystallization, the dual-polysaccharide interface, and beeswax oleogel is key to obtaining centrifugation-stable, crystal-sedimentation-free, high-load curcumin emulsions.

(2) Freeze–thaw stability

**Figure 7:**
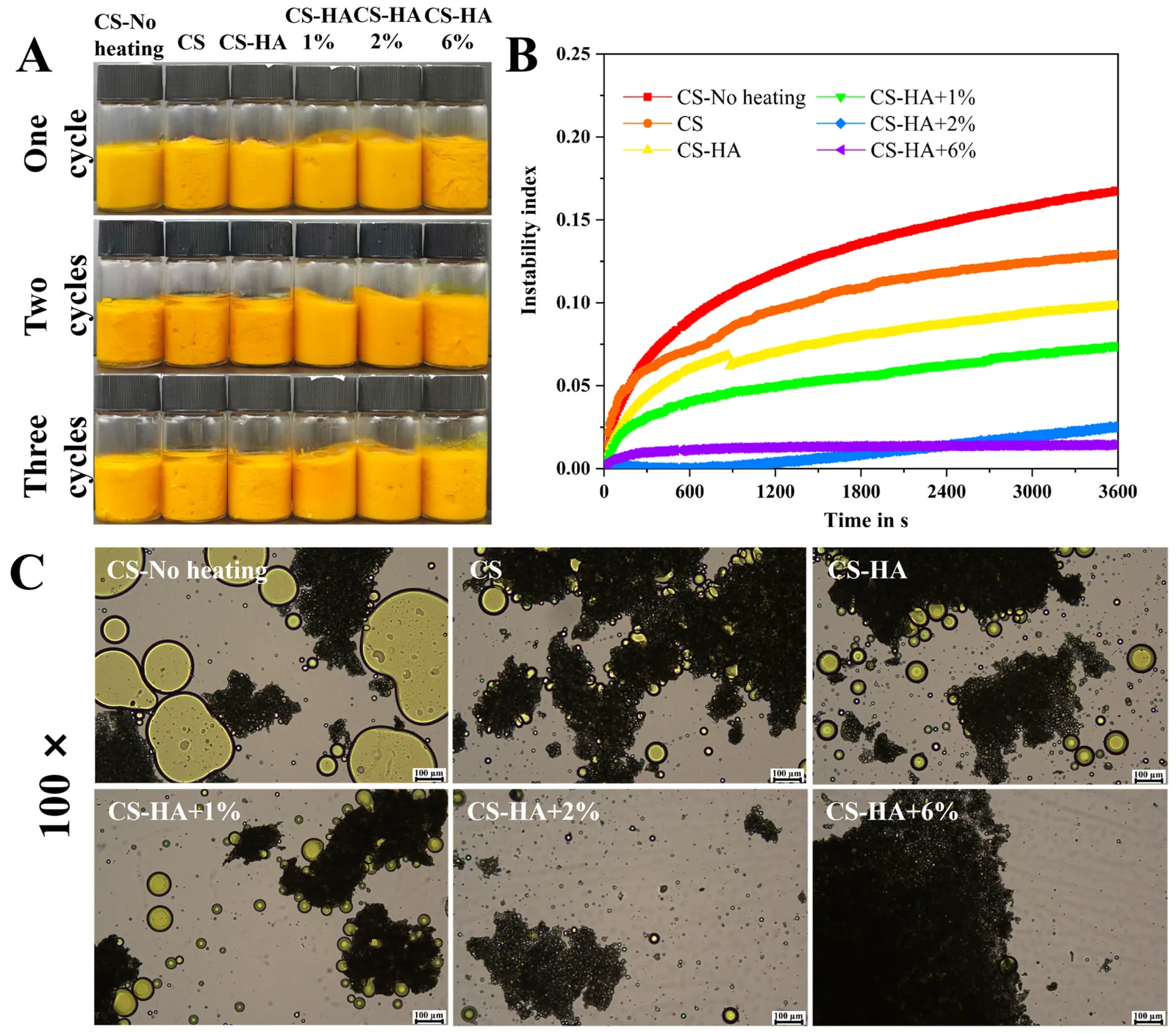
Freeze–thaw stability of the emulsions: Macroscopic appearance of the emulsions (**A**); time-dependent variation in the instability index (**B**); microstructure of the emulsions at 100× magnification (**C**).

Carriers loaded with active ingredients often require frozen storage for subsequent use; therefore, carriers with higher freeze–thaw stability and tolerance to more freeze–thaw cycles have broader application potential. As shown in Figure 7A, after three freeze–thaw cycles, both the single-polysaccharide and dual-polysaccharide emulsions without beeswax exhibited obvious upward floating of an oil layer, because ice crystals formed during freeze–thaw disrupted the oil–water interface, causing oil droplet leakage and coalescence. In contrast, the dual-polysaccharide emulsion with beeswax added to the oil phase showed markedly less upper oil. The freeze–thawed emulsions were then subjected to centrifugal stability testing, and the instability indices after 1 h were: CS-No heating (0.168), CS (0.129), CS-HA (0.098), CS-HA + 1% (0.074), CS-HA + 2% (0.025), and CS-HA + 6% (0.014) (Figure 7B). The microscopic morphology of the freeze–thawed emulsions was observed at 100× magnification (Figure 7C). The emulsion without heated oil-phase curcumin showed more large oil droplet coalescence. The heated single-polysaccharide and dual-polysaccharide aqueous-phase-stabilized emulsions without beeswax and with a low beeswax concentration (1%) all exhibited some oil droplet leakage and aggregation, whereas the dual-polysaccharide emulsions with higher beeswax concentrations (2% and 6%) showed less large oil droplet aggregation. It can therefore be concluded that emulsions prepared by heating had better freeze–thaw stability than high-load curcumin emulsion gels prepared without heating; dual-polysaccharide emulsions had better freeze–thaw stability than single-polysaccharide emulsions; and emulsions containing beeswax and a higher beeswax concentration (stronger gel strength) were more resistant to freeze–thaw cycles. The reasons are as follows: unheated emulsions contained more large curcumin crystals and had relatively fragile emulsion interfaces, so ice crystals accelerated interfacial instability during freezing [42]. Dual-polysaccharide interfacial emulsions had higher aqueous phase viscosity, and their interfacial layer may have been thicker and less easily disrupted than that of single-polysaccharide emulsions. Emulsions containing internal beeswax underwent gelation of the oil phase, making it more difficult for frozen crystals to disrupt the emulsion interface and cause oil droplet aggregation [43].

### 3.5. In Vitro Digestion Analysis

**Figure 8:**
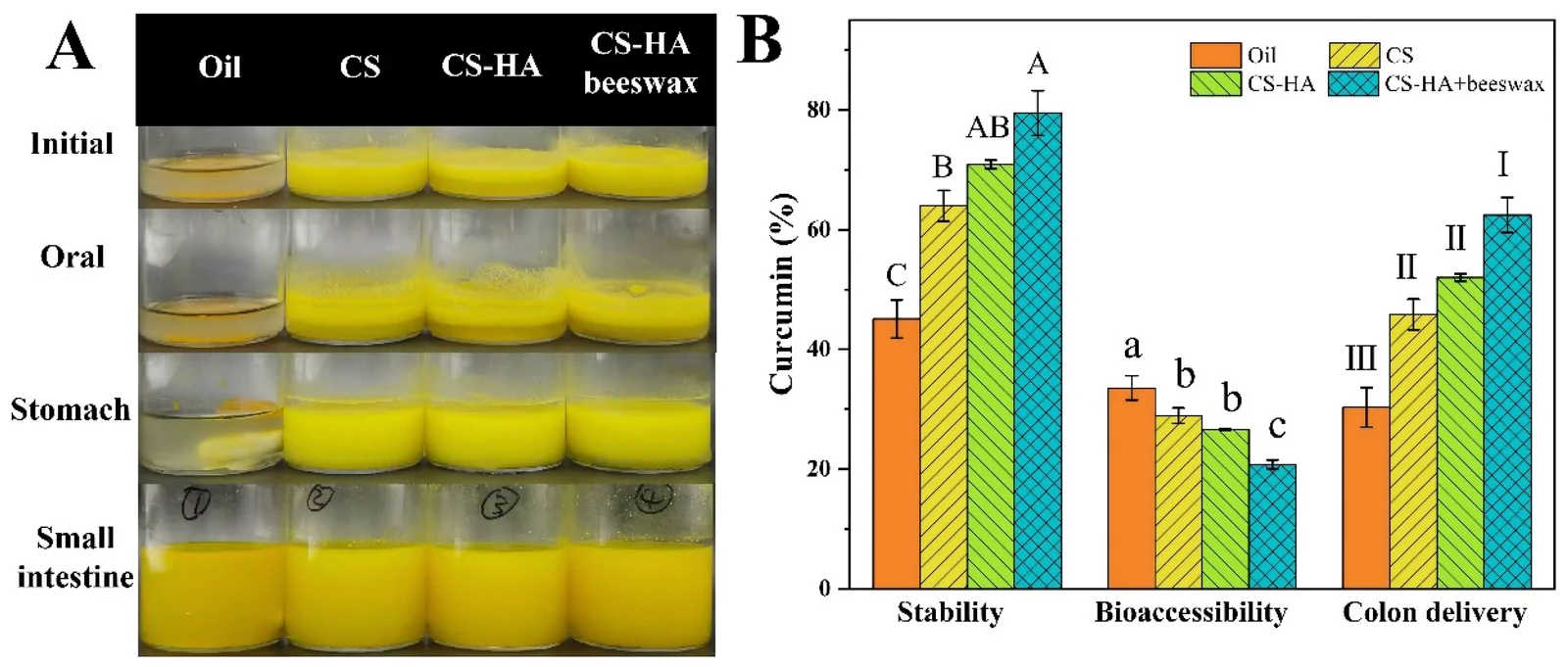
*In vitro* simulated digestion of oil and emulsions loaded with curcumin. Appearance of digestive fluid (**A**); stability, bioaccessibility, and estimated colon delivery of curcumin after intestinal digestion (**B**); different letters (A–C, a–c, and I–III) indicate significant differences within groups (*p* < 0.05).

The appearance of the digesta and key curcumin indicators (stability, bioaccessibility, and colon delivery) for four groups (Oil, CS, CS-HA, and CS-HA + beeswax (2%)) after *in vitro* simulated digestion were shown in Figure 8. Visual appearance (Figure 8A) showed that the curcumin-suspended oil group exhibited obvious oil–water separation at the initial and oral stages, whereas the three emulsions encapsulating curcumin and fish oil maintained a uniform yellow emulsion at the oral and gastric stages, with no obvious separation or oil droplet aggregation; at the small intestinal stage, all four groups appeared as bright yellow emulsions. Figure 8B shows that curcumin digestive stability was 45.10 ± 3.19% for Oil, 63.99 ± 2.60% for CS, 70.93 ± 0.71% for CS-HA, and 79.49 ± 3.74% for CS-HA + beeswax, with the beeswax dual-polysaccharide emulsion group significantly higher than the other groups (A); bioaccessibility was 33.54 ± 2.02% for Oil, 28.90 ± 1.29% for CS, 26.61 ± 0.09% for CS-HA, and 20.77 ± 0.76% for CS-HA + beeswax, with the beeswax dual-polysaccharide emulsion group significantly the lowest (c); and estimated colon delivery was 30.30 ± 3.29% for Oil, 45.85 ± 2.58% for CS, 52.02 ± 0.59% for CS-HA, and 62.45 ± 2.93% for CS-HA + beeswax, with the beeswax dual-polysaccharide emulsion group significantly the highest (I).

These results corroborate the colon-targeted delivery design of the synergistic polysaccharide interface and oleogel system. Free oil showed the lowest curcumin stability, indicating that without protection, the curcumin oil phase has poor stability in the gastric acid environment; in contrast, the three encapsulated emulsions, protected by polysaccharide interfacial films, maintained a uniform emulsified state at the oral and gastric stages, demonstrating good gastrointestinal structural stability. In terms of curcumin retention, the CS-HA + beeswax group had the highest digestive stability (79.49 ± 3.74%), significantly higher than free oil (45.10 ± 3.19%), the single-chitosan group, and the dual-polysaccharide group, which is attributed to the physical barrier of the polysaccharide interface and the confinement of curcumin crystals by the oleogel network, protecting them from degradation in the gastric and small intestinal digestive environments. Correspondingly, this group had the lowest small intestinal bioaccessibility (20.77 ± 0.76%, significantly lower than Oil, CS, and CS-HA) and the highest estimated colon delivery (62.45 ± 2.93%, significantly higher than Oil, CS, and CS-HA), indicating that the oleogel network effectively inhibited the dissolution and micellar absorption of curcumin at the small intestinal stage, thereby retaining more curcumin and delivering it to the colon. This “high stability–slow small intestinal absorption–high colon delivery” relationship is precisely the core feature of colon-targeted systems [26]. In summary, the emulsion with CS-HA and beeswax oleogel, through the synergy of the polysaccharide interface and oil-phase gel, significantly improved the gastrointestinal stability (by approximately 34%) and estimated colon delivery efficiency (by approximately 32%) of curcumin. A limitation of this evaluation is that the colonic stage was not modeled. The estimated colon delivery efficiency should thus be interpreted as a predictive indicator of colonic delivery potential, and actual colonic release and microbiota-mediated metabolism require further verification using colonic fermentation models or *in vivo* animal studies.

### 3.6. Oxidative Stability

To investigate the effects of aqueous-phase composition and oil-phase beeswax addition on fish oil oxidation, curcumin-free emulsions of different compositions and the oil phase (fish oil:coconut oil = 1:2) were simultaneously subjected to accelerated storage, and lipid oxidation was assessed at different time points (0, 7, and 14 days). The CS-HA + 2% formulation was selected for the oxidation test because 2% (*w*/*w*) was identified in the preceding sections as the optimal beeswax concentration, providing a uniform droplet distribution (unimodal peak) and significantly improved freeze–thaw stability, compared with the insufficient gelation at 1% and the potentially excessive hardness at 6%. This design was used to exclude the confounding antioxidant effect of curcumin and to evaluate the intrinsic protection provided by the interfacial and oleogel structures.

**Figure 9:**
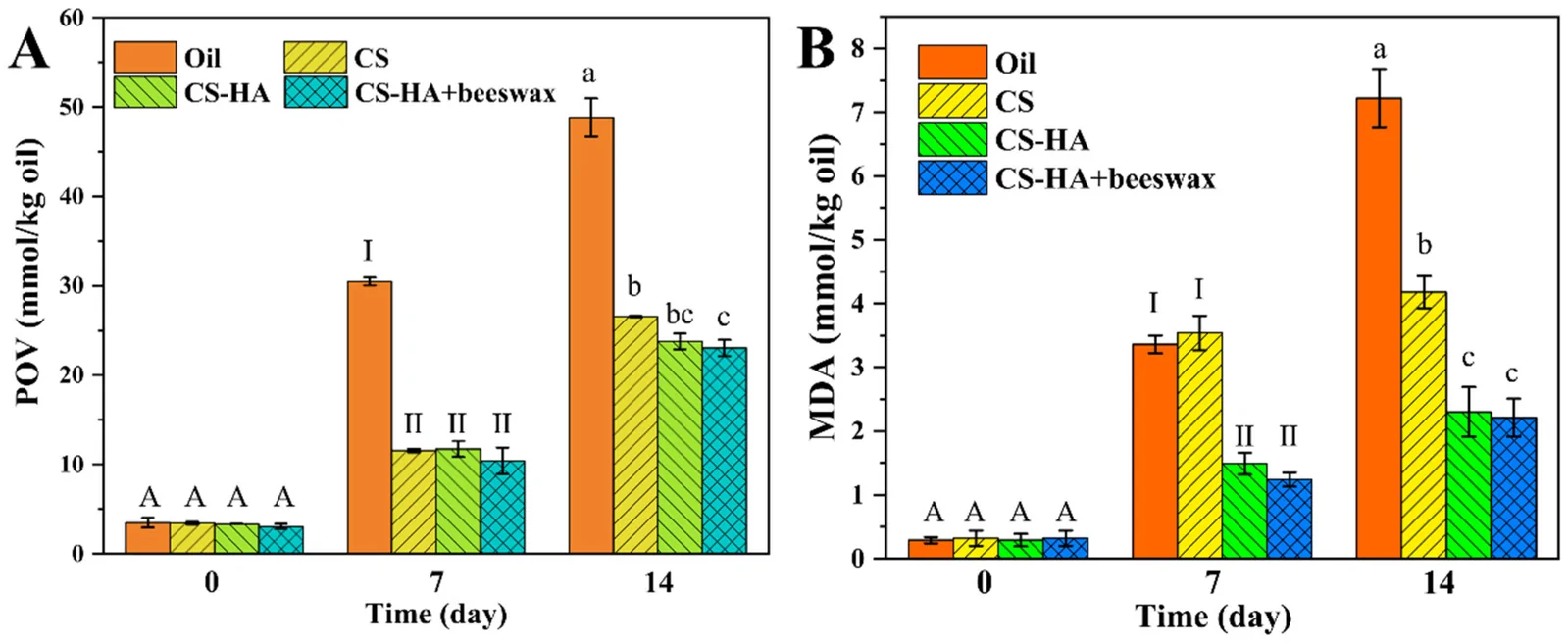
Primary oxidation products (POV) (**A**) and secondary oxidation products (MDA) (**B**) produced by the samples during the 14-day storage process (45 °C); different letters (A, a–c, and I–II) indicate significant differences within groups (*p* < 0.05).

Figure 9 showed the changes in the primary oxidation product peroxide value (POV) and the secondary oxidation product malondialdehyde (MDA) of emulsions with different compositions and the free oil phase (fish oil:coconut oil = 1:2) during accelerated storage. As shown in Figure 9A, at the initial storage time (day 0), the primary oxidation product content in all groups was at a low level (oil phase, CS, CS-HA, and CS-HA + beeswax, approximately 3 mmol/kg oil), with no significant differences among groups, indicating that the emulsion preparation process itself did not cause obvious oxidation of fish oil. After 7 days of accelerated storage, primary oxidation products accumulated in all groups, but with significant differences among groups: the free oil phase reached as high as 30.48 ± 0.44 mmol/kg, whereas the CS emulsion was only 11.55 ± 0.20 mmol/kg, approximately 62% lower than the oil phase; the CS-HA emulsion decreased to 11.73 ± 0.89 mmol/kg; and the CS-HA + beeswax emulsion with 2% beeswax was 10.41 ± 1.49 mmol/kg. After storage was extended to 14 days, POV continued to accumulate in all groups. The free oil phase rose to 48.84 ± 2.14 mmol/kg, while the CS, CS-HA, and CS-HA + beeswax groups were 26.58 ± 0.10, 23.77 ± 0.88, and 23.03 ± 0.92 mmol/kg, respectively. The emulsion groups remained significantly lower than the free oil phase, and the beeswax-containing dual-polysaccharide emulsion group was significantly lower than the single-polysaccharide emulsion group.

MDA remained at a relatively low level throughout the storage period, and the trend among groups was generally consistent with that of POV (Figure 9B). On day 0, MDA was close to zero, with no significant differences among groups. After 7 days of storage, the secondary oxidation product of the free oil phase was 3.36 ± 0.14 mmol/kg, showing no significant difference from that of the CS group (3.54 ± 0.27 mmol/kg), while the CS-HA and CS-HA + beeswax groups further decreased to 1.49 ± 0.17 and 1.24 ± 0.11 mmol/kg, respectively, with the CS-HA + beeswax group exhibiting the lowest mean value. After 14 days of storage, the secondary oxidation products of each group were as follows (oil phase: 7.22 ± 0.46, CS: 4.18 ± 0.25, CS-HA: 2.30 ± 0.39, CS-HA + beeswax: 2.21 ± 0.30 mmol/kg), overall still maintaining a decreasing trend of “free oil phase > CS > CS-HA ≈ CS-HA + beeswax”.

These results indicate that emulsification itself exerts a significant protective effect against fish oil oxidation, and that the dual-polysaccharide interface and oil-phase gelation further enhance this effect. In the free oil phase, unsaturated lipids are directly exposed to oxygen and pro-oxidant metal ions, rapidly driving the oxidative chain reaction, with POV exceeding 30 mmol/kg by day 7. In the emulsion system, fish oil is partitioned within droplets. The cationic polyelectrolyte adsorption layer formed by chitosan at the oil–water interface constitutes a physical barrier, while its positive charge can electrostatically repel pro-oxidant metal ions such as Fe^2+^ and Cu^2+^, and chitosan molecules themselves possess certain free radical scavenging capacity [44]. These multiple effects reduced the primary oxidation products of the CS emulsion by nearly two-thirds compared with the free oil phase.

Following the addition of hyaluronic acid, the secondary oxidation of emulsion lipids further decreased in all groups (at day 7, the secondary oxidation products decreased from 3.36 to 1.49 mmol/kg, a reduction of approximately 56%). This is because hyaluronic acid and chitosan, which is adsorbed at the interface, form a thicker and denser dual-polysaccharide interfacial composite layer through electrostatic interactions, extending the diffusion path of oxygen and pro-oxidant factors to the oil–water interface; meanwhile, hyaluronic acid also possesses the ability to scavenge free radicals and chelate metal ions [45]. Notably, the decrease in secondary oxidation products in the dual-polysaccharide group was much greater than that in primary oxidation, suggesting that the dense interfacial film not only slowed the formation of hydroperoxides but also more effectively inhibited their decomposition and propagation into secondary products such as aldehydes—the long-term low level of secondary oxidation products indicates that the accumulation of pro-oxidative decomposition products (polar compounds) in the lipid system itself was low, and the oxidative autocatalytic effect was weak [46].

Beeswax oleogel had a limited effect on further improving oxidative stability (no significant difference between the CS-HA + beeswax and CS-HA groups). It is speculated that the rate of primary oxidation is mainly dominated by the interfacial barrier; once the dual-polysaccharide interfacial film is established, the marginal contribution of oil-phase gelation to hydroperoxide formation is reduced. Therefore, at the selected 2% (*w*/*w*) beeswax concentration, the internal oleogel structure does not exert adverse effects on lipid oxidation. Although the oxidation experiment was performed on curcumin-free emulsions, these samples had the same structural compositions as the corresponding curcumin-loaded emulsions. Therefore, the oxidation results can be mechanistically correlated with the encapsulation and digestion data. The CS-HA + beeswax formulation, which maintained a high curcumin encapsulation efficiency (>95%, Figure 1C) and showed the highest digestive stability (79.49%) and colon delivery efficiency (62.45%) (Figure 8), also exhibited the lowest mean POV and MDA values among the emulsion groups during storage (Figure 9). This consistency indicates that the dual-polysaccharide interfacial barrier and the beeswax oleogel network simultaneously protect fish oil against oxidation and preserve curcumin within the delivery system. The dual-polysaccharide interface was the main contributor to oxidation protection, whereas the beeswax oleogel mainly contributed to crystal confinement, storage stability and colonic retention, without exerting an adverse effect on lipid oxidation. Because lipid oxidation products can compromise interfacial integrity and accelerate bioactive degradation, the lower oxidation in the CS-HA + beeswax system is expected to help maintain encapsulation and digestive stability.

## 4. Conclusions

In this study, a polysaccharide-interface-stabilized, high-load curcumin colon delivery emulsion gel was constructed based on a synergistic strategy combining heat-induced confined recrystallization within droplets and beeswax oleogel modulation. After the oil phase was heated and melted, emulsified, and rapidly cooled, curcumin recrystallized within droplets under the confinement of the interfacial film, avoiding disruption of the oil–water interface by native large crystals. The combined confinement of the dual-polysaccharide (chitosan–hyaluronic acid) interface and the beeswax crystalline network gave all heated emulsions encapsulation efficiencies above 95%, and droplets became finer and more uniform with increasing beeswax concentration. Rheological results indicated that all emulsions exhibited shear-thinning, gel-like behavior (G′ > G″), with viscosity and moduli increasing upon introduction of hyaluronic acid and increasing beeswax concentration. Centrifugation and freeze–thaw stability tests further confirmed that the beeswax oleogel network effectively suppressed interfacial disruption and oil droplet aggregation (the instability index after freeze–thaw decreased from 0.098 to 0.014). *In vitro* simulated digestion showed that the system exhibited colon delivery characteristics of “high digestive stability (79.49%), low small intestinal bioaccessibility (20.77%), and high colon delivery efficiency (62.45%).” Meanwhile, the polysaccharide interface and emulsion gel structure significantly delayed hydroperoxide formation and MDA accumulation in fish oil. Among the tested formulations, the CS-HA emulsion containing 2% (*w*/*w*) beeswax (CS-HA + 2%) was identified as the optimal formulation, as it balanced uniform microstructure, excellent freeze–thaw stability, the highest digestive stability/estimated colon delivery, and effective oxidative protection without excessive gel hardness.

However, this study has several limitations. First, the INFOGEST protocol simulates only the oral, gastric, and small intestinal phases; the colonic phase (including microbiota-mediated fermentation and actual colonic release) was not modeled, meaning the estimated colon delivery efficiency reported here is a predictive estimate. Second, the current findings are based on *in vitro* models, and *in vivo* animal studies are still required to confirm the actual bioavailability, metabolic fate, and colonic targeting efficacy of curcumin. Future work will focus on incorporating colonic fermentation models and *in vivo* animal experiments to further validate and optimize this delivery system for functional food applications. These results support the working hypothesis that the synergistic design of heat-induced confined recrystallization, a dual-polysaccharide interface, and an oleogel network can achieve high-load curcumin stabilization while simultaneously providing fish oil oxidation protection and potential colon-targeted delivery. This strategy provides a basis for designing food-grade, high-load bioactive oil colon delivery systems with potential for nutritional delivery and functional foods.

## Figures and Tables

**Table 1:** Particle size (D_3,2_ and D_4,3_) of the emulsions. Data were presented as mean ± SD.

Samples	D_3,2_ (μm)	D_4,3_ (μm)
CS-No heating	17.73 ± 0.15 ^a^	215.67 ± 2.52 ^a^
CS	11.30 ± 0.69 ^b^	196.67 ± 3.51 ^b^
CS-HA	5.88 ± 0.07 ^cd^	22.27 ± 0.90 ^d^
CS-HA + 1%	6.49 ± 0.10 ^c^	44.43 ± 5.50 ^c^
CS-HA + 2%	5.63 ± 0.02 ^d^	10.07 ± 0.06 ^e^
CS-HA + 6%	4.57 ± 0.02 ^e^	7.71 ± 0.08 ^e^

Different superscript letters in the same column indicate a significant difference (*p* < 0.05).

**Table 2:** K (consistency index), n (flow behavior index), and R^2^ obtained from the Power Law model based on the shear rate–viscosity curves of different emulsions.

Sample	K (Pa·s^n^)	n	R^2^
CS-No heating	9.574 ± 0.193	0.3246 ± 0.0107	0.9980
CS	10.859 ± 0.342	0.2237 ± 0.0164	0.9967
CS-HA	11.803 ± 0.417	0.1959 ± 0.0182	0.9963
CS-HA + 1%	13.333 ± 0.414	0.2068 ± 0.0160	0.9970
CS-HA + 2%	20.176 ± 0.354	0.2547 ± 0.0092	0.9989
CS-HA + 6%	38.128 ± 0.521	0.2731 ± 0.0072	0.9993

## Data Availability

The original contributions presented in the study are included in the article; further inquiries can be directed to the corresponding author.

## Abbreviations

The following abbreviations are used in this manuscript:

CS	chitosan
HA	hyaluronic acid
POV	peroxide value
MDA	malonaldehyde
